# A woman with recurrent umbilical bleeding: a case report

**DOI:** 10.1186/s13256-022-03675-2

**Published:** 2022-11-27

**Authors:** Yong-hun Kim, Abigail K. Wegehaupt, Majken T. Wingo

**Affiliations:** 1 Mayo Clinic Alix School of Medicine, Mayo Clinic, Rochester, MN USA; 2 Department of Medicine, Mayo Clinic, Rochester, MN USA; 3 Division of Community Internal Medicine, Geriatrics, and Palliative Care, Mayo Clinic, 200 First Street, SW, Rochester, MN 55905 USA

**Keywords:** Umbilical discharge, Umbilical bleeding, Umbilical hernia, Case report

## Abstract

**Background:**

Umbilical discharge in an adult is rare and generates broad diagnostic considerations. Umbilical anatomy is variable owing to congenital abnormalities and acquired pathology such as umbilical hernias. The umbilicus can be a site of primary or metastatic malignancy or endometriosis.

**Case presentation:**

A 40-year-old white American woman came to the clinic with a 2-day history of spontaneous umbilical bleeding. She reported periumbilical pain associated with nausea and emesis. There were no visible skin abnormalities, but deep palpation of the abdomen produced a thin, watery, serosanguineous fluid from the umbilicus. She experienced a similar episode of umbilical bleeding 5 years prior without clear cause. Laboratory workup was notable for mildly elevated C-reactive protein . Computed tomography imaging revealed a fat-containing umbilical hernia with fat necrosis, necessitating complete surgical resection of the umbilicus.

**Conclusions:**

Umbilical hernia with fat necrosis is a rare condition that should be considered in adults with umbilical discharge. Additional diagnostic considerations in adults with spontaneous umbilical bleeding/discharge include embryonal remnants, omphalitis, and metastasis. If the cause is not readily apparent on physical exam, imaging with computed tomography should be considered to assess for hernia and embryonal anomalies.

## Background

The rare case of spontaneous umbilical discharge in the adult patient presents differential diagnostic considerations relating to remnant embryologic structures, abdominal hernia, underlying medical conditions, infection, and foreign body material [1–4]. The case of spontaneous umbilical bleeding in the adult patient presents an even narrower differential with a scarcity in the available literature. Sarantitis *et al*. reported a case of umbilical bleeding caused by an incarcerated umbilical hernia with omental varices due to portal hypertension [2]. Tull *et al*. reported a case of a bleeding umbilical nodule due to cutaneous endometriosis [5]. We present a case of recurrent umbilical bleeding in an adult woman who was found to have a fat-containing umbilical hernia with fat necrosis after computed tomography (CT) and histopathologic examination. This is an uncommon cause of umbilical discharge that, to our knowledge, has not been previously reported in the literature.

## Case presentation

A 40-year-old white American woman presented to primary care with a chief concern of nontraumatic umbilical bleeding that began 2 days prior; she woke up and noticed her shirt soaked in blood from her umbilicus. Upon applying pressure with a rag, she was able to stop the bleeding. The patient continued to experience intermittent umbilical bleeding associated with nonradiating periumbilical pain that was exacerbated by movement and associated with nausea and emesis. She had been seen for non-painful umbilical bleeding 5 years prior when she had been occasionally cleaning her umbilicus in the shower with a cotton swab and peroxide; this would sometimes leave small spots of blood on the cotton swab. At that time, no imaging was performed; she was prescribed bacitracin and told to apply Vaseline for what was presumed to be superficial irritation. She had no interim symptoms and discontinued cleaning her umbilicus with a cotton swab.

Past medical history included hypertension, gastroesophageal reflux disease, class III obesity with BMI 45, major depression, and generalized anxiety. Patient is a G1P1001. Surgical history was notable only for elective caesarean section 12 years prior without complications. The patient was divorced and worked in retail. She did not smoke, use illicit drugs, or drink alcohol. Family history included diabetes, Crohn’s disease, diverticulitis, and breast, lung, and prostate cancer. The patient was taking multiple long-term prescription medications, including losartan 50 mg tablet by mouth daily, propranolol 60 mg tablet by mouth twice daily, escitalopram 20 mg tablet by mouth daily, bupropion 450 mg tablet by mouth daily, gabapentin 300 mg capsule by mouth three times daily, norethindrone-ethinyl estradiol 1 mg–35 µg tablet by mouth daily, trazodone 100 mg tablet by mouth daily, and diclofenac 1% gel topically four times daily as needed.

On the day of presentation, the patient’s blood pressure was 125/81 mmHg with a pulse of 65 beats per minute. She was afebrile. Inspection revealed a non-distended abdomen and completely normal skin without erythema, fissuring, or visible discharge, though there was some dried blood. There was mild periumbilical tenderness with deep palpation. Deep palpation around the umbilicus produced a thin, watery, serosanguinous fluid directly from the umbilicus. The remainder of the physical examination including cardiac, pulmonary, and neurologic examinations, which were normal.

**Fig. 1:**
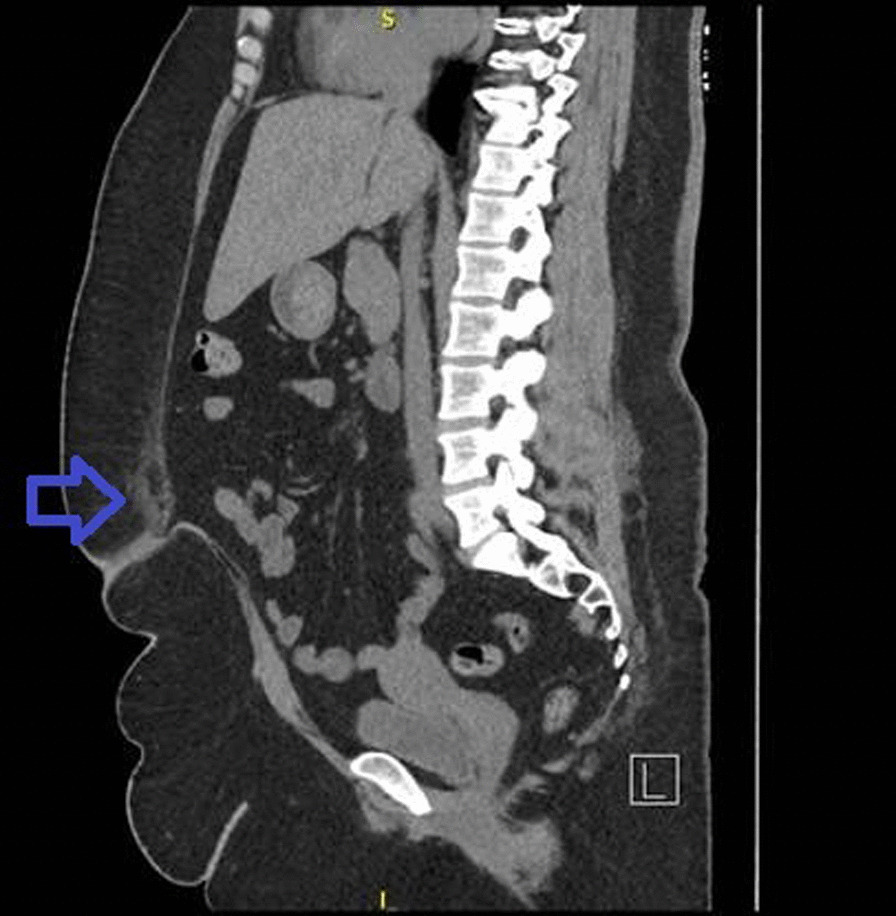
Sagittal computed tomography of the abdomen revealing an umbilical hernia (blue arrow) with fat superior to the umbilicus and hernia

**Fig. 2:**
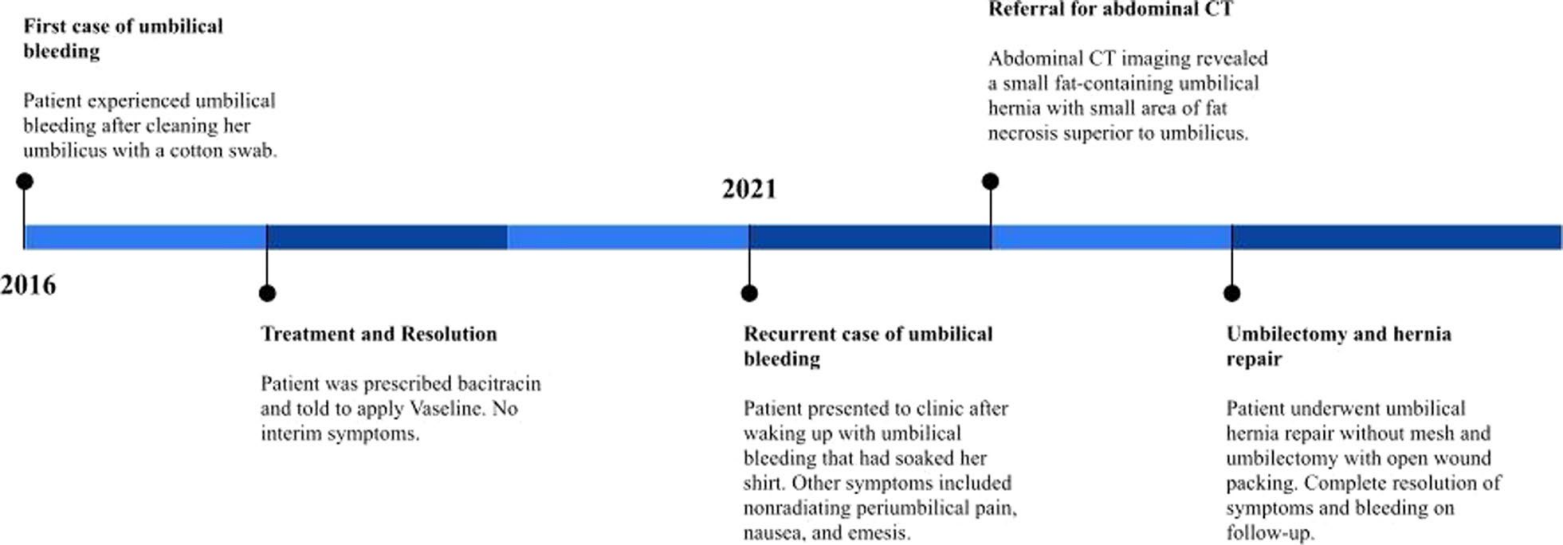
Timeline of patient’s history and care relating to both incidences of umbilical bleeding

The patient was referred for CT of the abdomen/pelvis the same day, which revealed a small fat-containing umbilical hernia with a likely small area of fat necrosis just superior to the umbilical hernia (Fig. 1). Laboratory workup was notable for a mildly elevated high-sensitivity CRP at 13.8. The remainder of labs, including complete blood count (CBC) with differential, electrolytes, renal function, and liver function were within normal limits. No coagulation parameters were checked. Four weeks later, the patient underwent outpatient open umbilical hernia repair without mesh and umbilectomy with open wound packing. Dissection was performed down to the level of the fascia, and a 4.2-by-3.5-by-2.6 cm specimen consisting of the hernia sac and urachal remnants was excised and sent for pathologic interpretation. No cultures were sent  since there was low suspicion for infection. She had complete resolution of symptoms and bleeding on follow-up 30 days postoperation. She did not receive any antibiotics or other prescription medications for this condition. Surgical pathology interpretation revealed ulcerated skin with abscess, umbilical remnant, granulation tissue, and foreign body suture material. At 1-month and 2-month wound check visits, the patient denied any pain, nausea, or vomiting. At next follow-up 6 months postoperation, the patient continued to do well without recurrence of her symptoms. A timeline of the patient’s history and care is presented in Fig. 2.

## Discussion

This case represents a rare presentation of an umbilical hernia that caused spontaneous bleeding from the umbilicus, which to our knowledge has not previously been reported. Spontaneous umbilical discharge in the adult patient is uncommon, and the diagnostic considerations are broad. Other case reports have characterized etiologies such as infection due to foreign body and pathologies associated with embryologic structures such as mullerianosis and umbilical sinus communicating with peritonitis [1–5]. One potentially life-threatening cause of bleeding can occur in the case of umbilical hernia with portal hypertension and rupture of umbilical varices that arise from recanalized umbilical and periumbilical veins or communication between underlying omental varices and abdominal wall scars [6, 7]. Though CT imaging revealed an umbilical hernia in our patient, there was an absence of cirrhosis and any abnormal vasculature that could imply rupture of umbilical varices. Endometriosis with umbilical implantation and primary malignancy or metastasis could also cause bleeding, but our patient’s pathology did not show endometrial tissue or malignancy [8].

Our patient had a history of cleaning her umbilicus with a cotton swab, which could potentially cause foreign body-induced omphalitis. Sarma *et al*. previously reported a case of omphalitis caused by an impacted lint ball [1]. Our patient’s first incidence of umbilical bleeding 5 years ago may have been caused by a similar foreign body with obesity and deep umbilicus constituting contributing risk factors. However, there was no precipitating event to cause her second case of umbilical bleeding, and physical exam did not reveal a foreign body or skin inflammation. Without an identifiable trigger, the bleeding is presumed to have been spontaneous. The patient’s only risk factor for umbilical hernia was elevated BMI. The origin of the foreign suture body material noted on pathology was unable to be determined and likely presents an incidental finding from the patient’s previous C-section.

## Conclusions

Umbilical discharge is an unusual condition caused by a wide range of pathology with varying degrees of severity. Umbilical hernia with fat necrosis should be considered when formulating a differential diagnosis.

## Data Availability

All data generated or analyzed during this study are included in this published article.
